# Clinical MEN-1 Among a Large Cohort of Patients With Acromegaly

**DOI:** 10.1210/clinem/dgaa142

**Published:** 2020-04-20

**Authors:** Lisa B Nachtigall, Francisco J Guarda, Kate E Lines, Alireza Ghajar, Laura Dichtel, Giselle Mumbach, Wenxiu Zhao, Xun Zhang, Nicholas A Tritos, Brooke Swearingen, Karen K Miller, Rajesh V Thakker

**Affiliations:** 1 Neuroendocrine Unit, Massachusetts General Hospital and Department of Medicine, Harvard Medical School, Boston, Massachusetts; 2 Endocrinology Department and Center for Translational Endocrinology (CETREN), School of Medicine, Pontificia Universidad Católica de Chile, Santiago, Chile; 3 Academic Endocrine Unit, OCDEM, Radcliffe Department of Medicine, University of Oxford, Churchill Hospital, Oxford, UK

**Keywords:** acromegaly, hyperparathyroidism, multiple endocrine neoplasia type 1 (MEN-1), MEN-1 phenocopy

## Abstract

**Context:**

Clinical multiple endocrine neoplasia type 1 (MEN-1) is diagnosed by the presence of at least 2 MEN-1–associated tumors. Many patients with acromegaly and clinical MEN-1 yield negative testing for *MEN1* mutations. While cases of acromegaly and primary hyperparathyroidism (PHP) with negative genetic testing have been reported, its prevalence among patients with acromegaly is undetermined, and the clinical presentation has not been well characterized.

**Objectives:**

The main goals of this study are: (1) To determine the prevalence of clinical MEN-1 with PHP in patients with acromegaly and characterize their clinical features; and (2) to evaluate the genetic basis for the coexistence of acromegaly and PHP.

**Design:**

Retrospective record review and genetic analysis.

**Setting:**

Clinical Research Centers.

**Participants:**

414 patients with acromegaly.

**Interventions:**

Clinical evaluation and DNA sequencing for *MEN1*, *CDKN1A*, *CDKN1B*, *CDKN2B*, *CDKN2C,* and *AIP* genes.

**Main outcome measurements:**

Clinical and genetic analysis.

**Results:**

Among patients with acromegaly, clinical MEN-1, as defined by the presence of at least one other MEN-1-associated tumor, was present in 6.6%. PHP occurred in 6.1%; more than half had parathyroid hyperplasia. DNA sequencing was unrevealing for genetic mutations, except for 1 case of a *CDC73* mutation. Acromegaly was diagnosed at an older age with a higher prevalence of malignancies (specifically breast and thyroid) in patients with coexisting PHP than those with isolated acromegaly.

**Conclusions:**

A distinct phenotype is described in patients with clinical MEN-1 and negative genetic testing for mutations previously associated with this syndrome. Further studies are needed to identify other genes that may explain the association between PHP and acromegaly.

Multiple endocrine neoplasia type 1 (MEN-1) is an autosomal dominant disorder characterized by parathyroid, pituitary, and entero-pancreatic neuroendocrine tumors (NETs). Clinical MEN-1 may be defined by the combined presence of 2 of these endocrine tumors, or at least one in addition to a first degree relative with confirmed MEN-1 (1, 2). The coexistence of acromegaly and primary hyperparathyroidism (PHP) in 1 individual is therefore considered diagnostic of clinical MEN-1 (1, 3). However, in 5% to 25% of patients with a MEN-1 phenotype, genetic testing is negative for *MEN1* mutations, which may be explained by mutations in as yet unidentified genes or may represent phenocopies (clinical phenotypes that mimic those associated with known genetic mutations, but which occur without the expected genotype) (2, 4, 5). With only pituitary and parathyroid tumors, the *MEN-1* mutation may be found with a lower frequency, even less common in sporadic cases (6).

Overall, pituitary involvement can be seen in 30% to 40% of MEN-1 patients, with growth hormone-secreting tumors representing approximately 5% to 25% of cases, as previously reported (1, 3, 7, 8). Among patients with isolated acromegaly, it has been shown that 1.2% of those younger than 30 years with sporadic cases can harbor a mutation in the *MEN1* gene (7). Previous studies have shown that among patients with pituitary adenomas of all subtypes, the prevalence of clinical MEN-1 is 4.8% to 7.7% (9, 10). However, the prevalence of clinical MEN-1 in a large cohort of patients with acromegaly has not been well described. In fact, in the descriptions of populations with pituitary adenoma, the prevalence of clinical MEN-1 within the subgroup with acromegaly has been limited to a small number of patients and ranges widely from 2.9% to 18.5% (9, 10). The prevalence of PHP in patients with acromegaly has not been specifically addressed in large cohorts but it would be expected to occur in 0.86% of the general population (11).

Many patients with clinical MEN-1 may have negative testing for *MEN1* mutations due to genetic abnormalities in noncoding regions or whole-gene deletions, detection of which requires more extensive DNA sequencing analysis (12). If the *MEN1* gene is excluded, other genes known to be associated with endocrine tumors may be involved. For example, MEN-4 was described as a distinct clinical entity caused by mutations in *CDKN1B,* which can present clinically as the coexistence of pituitary adenomas and PHP (13). Nevertheless, genetic studies in patients with clinical MEN-1 and negative *MEN1* mutations have shown a low frequency of *CDKN1B* pathogenic variants (14). Mutations within genes that regulate other functions and that have been associated with the occurrence of endocrine neoplasia, such as cell division cycle 73 (*CDC73*), and aryl hydrocarbon receptor-interacting protein (*AIP*), among others, may also be found among patients with parathyroid and/or pituitary tumors (4, 12, 13, 15).

The main goals of this study are: (1) to determine the prevalence of clinical MEN-1 and PHP in a large group of patients with acromegaly and characterize the clinical features of this subset; and (2) to evaluate the genetic basis for the coexistence of acromegaly and primary PHP.

## Methodology

**Figure 1.**
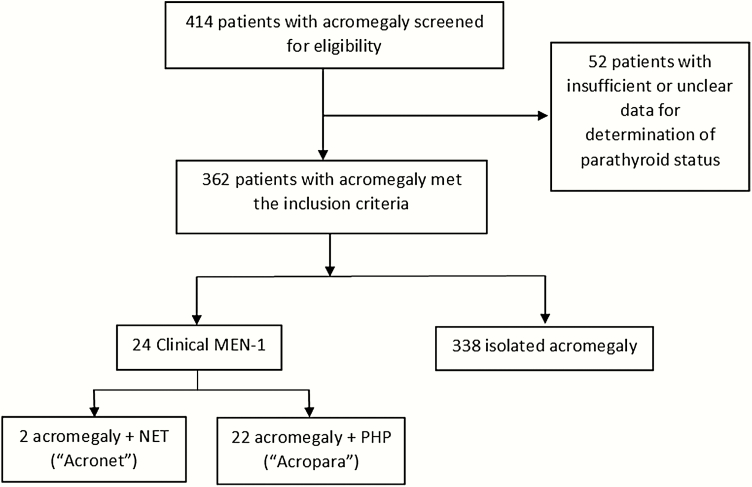
Subject selection flow diagram * 1 patient in the Acropara group developed a neuroendocrine tumor (patient 6 in Table 1).

This study was approved by the Institutional Review Board of Partner’s Healthcare. Records from an acromegaly database, including patients seen and surgically confirmed to have acromegaly at a single pituitary center from 1980 to 2019 (N = 414) were reviewed. Subjects were included in this study if serum calcium levels were available (n = 365). Patients with acromegaly were divided into 2 groups. Criteria for inclusion in the clinical MEN-1 group were biochemical and/or histopathologic evidence of PHP and/or NETs. Those with growth hormone (GH)-secreting tumors in the absence of other major features of clinical MEN-1 (NETs and PHP) were included in the isolated acromegaly group. Patients in whom biochemical or histopathologic parathyroid status was unclear (n = 3) or unavailable (n = 49) were excluded (Fig. 1). The clinical MEN-1 group was then subdivided into 2 groups according to the presence of PHP (Acropara, n = 22) or absence of PHP (Acronet, n = 2), as shown in Fig. 1. Personal history of malignancies was obtained from reports by attending clinicians and the prevalence of each disease was calculated among all individuals in each group. In patients with thyroid and adrenal tumors, information on neck ultrasound and abdominal imaging was retrieved and prevalences were also calculated in this specific subset of patients. Sixteen out of the 22 patients with PHP provided a blood sample with informed consent for genetic testing and were interviewed regarding their family histories. DNA sequence analysis was performed on genes including: *MEN1*, *CDC73*, *CDKN1A*, *CDKN1B*, *CDKN2B*, *CDKN2C*, and *AIP*. In addition, multiplex ligation-dependent probe amplification (MLPA) was used to search for deletions or duplications in *MEN1*, *CDC73*, *CDKN1B*, and *AIP* genes. Of the 6 patients within the acromegaly and PHP group who did not provide consent for DNA sequence analysis, 2 elected to have clinical genetic testing performed by a commercially available genetic screening test as per clinical consultation with the Endocrine Cancer Genetics Clinic at Massachusetts General Hospital. The 2 patients with acromegaly and NETs in the absence of PHP declined genetic testing and are described separately.

### DNA extraction and sequencing

Genomic DNA was extracted from human blood leukocytes and purified by using QIAamp DNA Blood Maxi Kit (Qiagen, MD). Leukocyte DNA was screened for mutations by fluorescent Sanger sequencing of the coding region and exon/intron boundaries of *MEN1, CDC73, CDKN1A, CDKN1B, CDKN2B, CDKN2C*, and *AIP*, and a 5′ UTR open reading frame (uORF) in *CDKN1B* (c.519-430) in which a MEN4 pathogenic mutation has been identified (16). The MLPA kit P244-C1 (MRC Holland) was used to detect deletions or duplications of exons of *MEN-1* (exons 1-11), *CDKN1B* (exons 1-3), and *AIP* (exons 1-6), and the MLPA kit P466-A1 was used to detect deletions or duplications of *CDC73* (exons 1-17 and 3′ UTR). Accession sequences: *MEN-1* (NM_130799.2); *CDC73* (NM_024529.4); *CDKN1A* (NM_000389.3); *CDKN1B* (NM_004064.3); *CDKN2B* (NM_004936.3); *CDKN2C* (NM_001262.2); *AIP* (NM_003977.2) as previously described (17). The 2 patients who underwent commercially available gene sequencing were assessed through protocols that involved PCR amplification of the 9 coding regions of the *MEN1* with flanking regions containing intron-exon junctions at Yale Biochem Disease Detection Lab and Gene Dx.

### Biochemical analyses

Insulin-like growth factor 1 (IGF-1) was measured using liquid chromatography-mass spectrometry (LC-MS) (Quest Diagnostics, San Juan Capistrano, CA) after 2013. Immulite 2000 immunoassay system by Siemens Medical Systems and Nichols Advantage were used from 2005 to 2013 and from 2003 to 2005, respectively. Other assays were used before 2003, such as LC-MS from Mayo, IRMAs, RIAs and ICMAs. Prolactin was measured using a competitive ELISA kit (ALPCO, Salem, NH). Different assays were used for the determination of plasma 25-hydroxyvitamin D depending on the time of the analysis, including radioimmunoassay (DiaSorin RIA), enzyme immunoanalysis (DiaSorin Liaison) and Liquid Chromatography/Tandem Mass Spectrometry (LC-MS/MS). Intact parathyroid hormone (PTH) levels were measured by different assays along time, the latest being electrochemiluminescence immunoassay, Roche Diagnostics.

Acromegaly was diagnosed based on IGF-1 index greater than 1, defined as IGF-1 level divided by the upper limit of normal for age and sex, associated with clinical features, a nonsuppressed GH after a 75 g glucose load and/or pathological confirmation after transsphenoidal surgery. The presence of PHP was determined in cases of PTH-dependent hypercalcemia or normocalcemia with elevated PTH, normal 25-hydroxy vitamin D levels and exclusion of other conditions causing hypercalcemia, such as familial hypocalciuric hypercalcemia. In all patients in whom 25-hydroxy vitamin D levels were low, supplementation was given and calcium, PTH, and 25-hydroxy vitamin D levels were reassessed. In patients who had adrenal adenomas, functional testing included serum renin and aldosterone levels, urinary or serum metanephrines, and dexamethasone suppression testing for cortisol excess.

### Statistical analysis

Statistical analyses were performed using Stata Statistical Software: Release 16 (College Station, TX: StataCorp LLC). Continuous variables were tested for normality using the Shapiro-Wilk test. Quantitative data were expressed as mean and standard deviation (SD) for those with Gaussian distributions, or as median and interquartile range (IQR) for those with non-Gaussian distributions. Categorical or dichotomous variables were compared using the Chi-square and Fisher exact tests, as appropriate. Differences in clinical characteristics between patients with clinical MEN-1 phenotype (i.e., acromegaly with PHP) vs isolated acromegaly were assessed by a 2-sample *t* test for normally distributed continuous variables or Wilcoxon rank-sum test for continuous variables that were non-normally distributed. In order to adjust for age as a potential confounder, multivariate logistic regression was performed when appropriate, and odds ratios (OR) are reported with their respective 95% confidence intervals. Statistical significance was defined as a 2-sided P value of less than 0.05.

## Results

### Clinical characteristics

#### Baseline clinical characteristics of patients with clinical MEN-1.

The prevalence of patients who demonstrated a phenotype of clinical MEN-1 (i.e., any ≥ 2 of the 3 major endocrine tumors associated with the MEN-1) was 6.6% (24/362). Among the 22 patients with acromegaly and PHP (Acropara group), 13 (59.1%) were female, 15 of the 19 (79.0%) who had data on tumor size available had pituitary macroadenomas. The age at diagnosis of acromegaly was 54.2 ± 15.9 years, and for PHP it was 60.5 ± 12.5 years. Acromegaly was diagnosed before or at the same time as PHP in 20/22 patients (90.9%). Regarding PHP, 13/22 patients (59.1%) had low bone mineral density, including 8 with osteoporosis (mainly at the femoral neck) and 5 with osteopenia. Kidney stones were clinically recognized in 4/22 (18.2%). Among the 6 patients who did not undergo parathyroidectomy, only 1 did not have elevated calcium levels, but rather had high-normal calcium with elevated PTH and normal 25-hydroxy vitamin D, consistent with normocalcemic hyperparathyroidism. The remaining 4 demonstrated hypercalcemia. When considering conditions that may be associated with the MEN-1 phenotype in a first-degree relative, such as PHP or hypercalcemia, pituitary tumors, and/or gastro-entero-pancreatic NETs, 2/22 patients (9.1%) had positive findings: patient 18 had a daughter with hypercalcemia and patient 19 had a father with acromegaly and sister with prolactinoma. Additionally, 6 patients had a family history of endocrine-related conditions, which might be associated with the MEN-1 phenotype, such as nephrolithiasis, non-neuroendocrine pancreatic tumors, and pituitary disease in second- or third-degree relatives. Clinical data on each of the patients in the clinical MEN-1 group are summarized in Table 1.

**Table 1.** Clinical and Genetic Characteristics of Patients with Clinical MEN-1

Patient	Sex	Age at Diagnosis (years)	Pathology	Malignancies	Family History of Malignancy	Mutation Analysis
Acropara		(Acro/PHP)	Parathyroid pathology			
1	F	57/49	Hyperplasia	No malignancy	Yes	Negative
2	F	59/78	Hyperplasia	No malignancy	No	Negative
3	M	65/70	Hyperplasia	Squamous cell carcinoma of the skin	Yes	Negative
4	F	19/35	Hyperplasia	No malignancy	Yes	Negative
5	M	57/57	Hyperplasia^a^	Renal cell carcinoma, Papillary thyroid cancer	No	Negative
6	M	24/70	NA	Pancreatic NET	Yes	CDC73 mutation
7	F	51/58	Hyperplasia	Breast cancer	Yes	Negative
8	F	34/38	NA	No malignancy	No	Negative
9	F	55/58	NA	Breast Cancer	No	Negative
10	F	44/52	Adenoma	No malignancy	Yes	Negative
11	F	31/45	Adenoma	No malignancy^b^	Yes	Negative
12	M	68/68	Hyperplasia	No malignancy	No	Negative
13	F	54/74	NA	Glioblastoma, Breast Cancer	No	Negative
14	F	46/49	Normocellular	Papillary thyroid cancer	Yes	Negative
15	F	71/71	NA	No malignancy	No	Negative
16	M	73/70	Adenoma	Basal cell and squamous cell carcinoma of the skin	Yes	Negative
17	M	61/61	Hyperplasia	Bladder cancer, Papillary thyroid cancer	Yes	Unknown
18	M	61/67	Hyperplasia	Prostate Cancer	Yes	Negative^c^
19	F	56/56	NA	B-cell lymphoma	No	Negative^c^
20	M	55/55	Double adenoma	No malignancy	Yes	Unknown
21	M	80/80	Adenoma	No malignancy	No	Unknown
22	F	71/71	Adenoma	No malignancy	Yes	Unknown
**Acronet**		**(Acro/NET)**	**NET pathology**			
23	M	49/63	Metastatic GI carcinoid	No other malignancies	No	Unknown
24	M	51/35	Bronchial carcinoid	No other malignancies	No	Unknown

Age at diagnosis for the Acropara group is shown for acromegaly and PHP. Age at diagnosis for the Acronet group is shown for Acromegaly and NETs.

Abbreviations: Acro, acromegaly; F, female; GI, gastrointestinal; M, male; NA, not available; NETs, neuroendocrine tumors; PHP, primary hyperparathyroidism.

^a^Based on outside hospital description of hyperplasia, official pathology not available.

^b^This patient presented a vaginal high grade squamous intraepithelial lesion, which is considered premalignant.

^c^These patients had commercially available MEN-1 genetic testing for menin, with no further analysis of other genes.

#### Endocrine tumor histopathology among patients with acromegaly and PHP (Acropara group).

All patients with Acropara had pituitary surgical therapy for acromegaly, including pathological confirmation of an adenoma. Concomitant staining for prolactin was positive in 10/15 (66.7%) with available immunostaining. Sellar radiation therapy was administered in 6/22 (27.3%). Parathyroid surgery was performed on 16/22 (72.7%) patients diagnosed with PHP. Parathyroid adenomas were found in 6 (37.5%), hyperplasia in 9 (56.3%), and 1 specimen showed normocellular tissue (Table 1). One patient presented with 2 simultaneous adenomas within the parathyroid glands. Adrenal imaging was available in 17 patients. Four of these patients (23.5%) had adrenal adenomas in whom 2 were bilateral. Among the 13 patients who had thyroid imaging available for analysis, 8 patients (61.5%) had thyroid nodules. Thyroid cancer was diagnosed in 3 patients. Malignancies were present in 11 patients, with breast cancer being the most frequent (23.1% of women), followed by papillary thyroid cancer (13.6% overall). One patient within the Acropara group developed a low grade pancreatic NET (patient 6 in Table 1). The details of this case and the pancreatic tumor were separately reported (17). Family history of malignancies was present in 13/22 patients (59.1%) and 2/22 (9.1%) also had a positive family history of pituitary disease.

#### Endocrine tumor histopathology among patients with acromegaly and NET (Acronet group).

Two patients with acromegaly (and no PHP) had NETs and refused genetic testing. These patients were included in the Acronet group (Fig. 1). One patient presented with acromegaly and a macroadenoma at age 49. He had 2 transsphenoidal surgeries but failed to go into remission. He developed an ileal NET with metastases to lymph nodes at age 63. He died at age 74. The cause of death was thought to be related to chronic heart failure and atrial fibrillation (patient 23, Table 1). The second patient had bronchial carcinoid diagnosed at age 35, which was surgically cured. He presented with acromegaly and a pituitary microadenoma at age 51. He underwent surgical therapy with transsphenoidal surgery which showed an adenoma with most tumor cells staining positive for GH and prolactin. He had renal cysts and kidney stones after age 50, with normal serum calcium and PTH. At last follow-up, bronchial carcinoid and acromegaly remained in remission (patient 24, Table 1).

#### Characteristics of patients with acromegaly and PHP (Acropara group) compared with isolated acromegaly.

**Figure 2.**
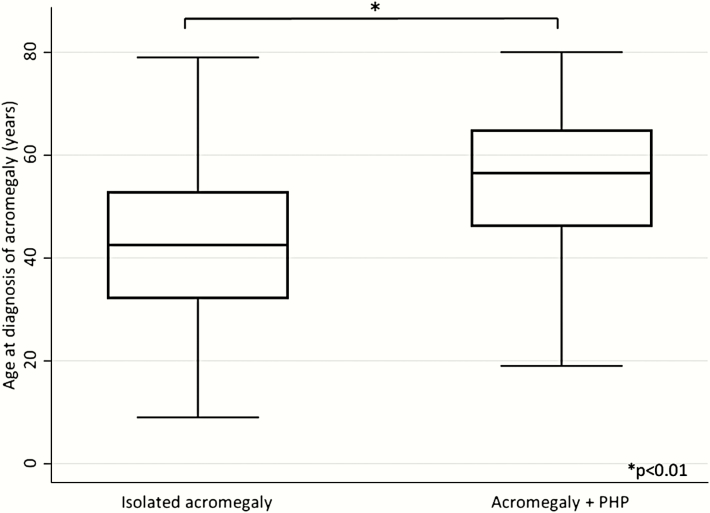
Age at diagnosis of acromegaly PHP: primary hyperparathyroidism.

Patients within the Acropara group (N = 22) were compared to those with isolated acromegaly (N = 338). As shown in Table 2, gender, IGF-1 index at diagnosis, and tumor size were not different between the 2 groups. Age at diagnosis of acromegaly was significantly higher among patients with PHP (Fig. 2 and Table 2). As expected by design, serum calcium and PTH levels were higher and phosphorus levels were lower in the Acropara group (Table 2). There were no differences in serum prolactin levels or IGF-1 index at diagnosis. Prolactin immunostaining of pituitary adenomas was positive in 10/15 (66.7%) in patients with Acropara and 207/272 (76.1%) in the isolated acromegaly group (*P* = 0.407). The age at last follow-up was significantly higher among patients in the Acropara group, while the duration of follow-up did not differ. The prevalence of normal IGF-1 levels at last follow-up was not different between Acropara and isolated acromegaly. There was no difference in therapeutic modalities, including repeated pituitary surgery or the use of radiation therapy between groups (Table 2).

**Table 2.** Characteristics of Patients with Clinical MEN-1 Versus Isolated Acromegaly

	Acropara (N=22)	Isolated Acromegaly (N=338)	*P* Value
**Clinical assessment**			
Female, n (%)	13 (59.1%)	186 (55.0%)	0.710
Age at diagnosis of acromegaly, years	54.2 ± 15.9	42.6 ± 14.0	**<0.001**
Pituitary macroadenoma, n (%)	15 (79.0%)	262 (81.4%)	0.810
Pituitary tumor maximum diameter at diagnosis, cm	1.5 [1.0-2.0]	1.5 [1.1-2.5]	0.337
Family history of pituitary disease, n (%)	2 (9.1%)	12 (3.7%)	0.224
Repeated surgery, n (%)	3 (13.6%)	45 (13.3%)	1.000
Radiation therapy, n (%)	6 (27.3%)	87 (25.7%)	0.810
IGF-1 normalization at last follow-up, n (%)	19 (90.5%)^a^	266 (85.4%)	0.750
Age at last follow-up, years	68.7 ± 11.8	52.8 ± 14.7	**<0.001**
Duration of follow-up, years	11.5 [4-17]	9.0 [2-15]	0.138
**Biochemical assessment**			
GH at diagnosis, ng/mL	13.8 [7.8-28.4]	10 [4.5-20.4]	0.233
IGF-1 index at diagnosis	2.5 [2.2-3.5]	2.3 [1.6-3.1]	0.153
IGF-1 index at last encounter	0.7 [0.4-0.8]	0.7 [0.5-0.9]	0.952
Prolactin level, ng/mL	12.2 [7.1-39.8]	12.1 [6.9-26.1]	0.964
Calcium level, mg/dL	11.0 ± 0.8	9.5 ± 0.5	**<0.001**
Phosphorus level, mg/dL	2.8 ± 0.5	3.6 ± 0.7	**<0.001**
Parathyroid hormone level, pg/mL	137 [78-160]	37 [26-53]	**<0.001**
25-hydroxy vitamin D level, ng/mL	28 [23-30]	33 [28-42]	0.091

Acropara includes the group of patients who had acromegaly and primary hyperparathyroidism. Results are shown as mean ± standard deviation or median [25^th^ and 75^th^ percentile] depending on data distribution.

^a^Information on normalization of IGF-1 at last follow-up was available for 21/22 patients.

^b^Several variables in the isolated acromegaly group were not available for all 338 patients given the retrospective nature of the analysis.

Among those with thyroid imaging available, no statistically significant difference in the prevalence of thyroid nodules was found between groups (8/13 [61.5%] patients in the Acropara group vs 75/97 [77.3%] in the isolated acromegaly group; *P* = 0.214). The overall prevalence of adrenal adenomas was higher in the Acropara cohort than in the isolated acromegaly group (18.2 vs 1.8%; *P* = 0.002), but the significance was lost after excluding the subset with unavailable abdominal computed tomography or magnetic resonance imaging (23.5 vs 8.8%; *P* = 0.1, respectively). Remarkably, only patients within the Acropara cohort presented with more than one adrenal adenoma. There was no evidence of adrenal hyperfunction in the Acropara group, all of whom had functional testing for adrenal hormone excess. In the isolated acromegaly group, a single patient had adrenal hyperfunction, in whom primary hyperaldosteronism was confirmed. Of the remaining patients with isolated acromegaly who had adrenal adenomas, none had clinical evidence of adrenal hormone excess.

#### Malignancies among patients with acromegaly and PHP (Acropara group) compared to isolated acromegaly.

The presence of any malignancy was higher among the Acropara group than in the isolated acromegaly cohort (50.0 vs 14.0%; *P* < 0.001), specifically for breast and thyroid cancer, as shown in Table 3. In order to assess the effect of age on malignancies between groups, multivariate logistic regression was performed and showed that the difference between groups remained statistically significant after adjustment for age (*P* = 0.023; OR 3.0 [1.2-7.9]). Breast and thyroid cancer were also more frequently present in patients among the Acropara group than in the isolated acromegaly group (23.1% vs 2.2%; *P* = 0.007 and 13.6% vs 2.7%; *P* = 0.031, respectively). In addition, multivariate logistic regression was performed to assess the effect of age in the frequency of each of these 2 types of cancer between groups. Breast cancer showed a significant difference between groups after adjustment for age (*P* = 0.018; OR 7.9 [1.4-43.5]). However, thyroid cancer prevalence was not significantly higher in Acropara, after adjustment for age (*P* = 0.059). The prevalence of thyroid cancer in patients who had available neck imaging (ultrasound, computed tomography or MRI) was 23.1% vs 9.3%; *P* = 0.151. Family history of malignancy was significantly higher in the Acropara group (59.1 vs 33.4%; *P* = 0.014). There were no differences between the Acropara and the isolated acromegaly groups regarding family history of pituitary disease (Table 2).

**Table 3.** Type of Malignancies in Patients With Clinical MEN-1 Versus Isolated Acromegaly

	Acropara	Isolated Acromegaly	*P* Value
Personal history of any malignancy	11/22 (50.0%)	47/336 (14.0%)^a^	**<0.001** ^**b**^
Breast cancer	3/13 (23.1%)	4/185 (2.2%)	**0.007** ^**b**^
Thyroid cancer	3/22 (13.6%)	9/335 (2.7%)	**0.031**
Skin cancer	2/22 (9.1%)	8/335 (2.4%)	0.121
Prostate cancer	1/9 (11.1%)	3/150 (2.0%)	0.210
Bladder cancer	1/22 (4.5%)	2/335 (0.6%)	0.174
Glioblastoma	1/22 (4.5%)	2/335 (0.6%)	0.174
Hematologic neoplasm^c^	1/22 (4.5%)	4/335 (1.2%)	0.274
Neuroendocrine tumor	1/22 (4.5%)	0/335 (0%)	0.062
Renal cell cancer	1/22 (4.5%)	4/335 (1.2%)	0.274
Colon cancer	0/22 (0%)	3/335 (0.9%)	1

Acropara includes the group of patients who had acromegaly and primary hyperparathyroidism.

^a^In the isolated acromegaly group, personal history of malignancy was available for 336 out of 338 patients. One female patient in the isolated acromegaly group had chemotherapy for a non-specified cancer. The other cancer types identified in the isolated acromegaly group included condrosarcoma, gastric sarcoma, testicular cancer and uterine cancers.

^b^Significant after multivariate logistic analysis

^c^Hematologic neoplasms include B-cell lymphoma, micosis fungoides, chronic lymphocytic leukemia and marginal cell lymphoma

#### DNA analysis in patients with acromegaly and PHP.

Sixteen patients with acromegaly and PHP underwent rigorous genetic testing with DNA sequence analysis performed for genes including: *MEN1*, *CDC73*, *CDKN1A*, *CDKN1B*, *CDKN2B*, *CDKN2C,* and *AIP*, as well as MLPA to search for deletions or duplications in *MEN1*, *CDC73*, *CDKN1B*, and *AIP* genes. A *CDC73* heterozygous missense mutation (Leu380Phe) was identified in a single patient within the Acropara group who presented with acromegaly, mild PHP, and developed a pancreatic NET, with no known family history of endocrine tumors. This case has been individually reported with detailed pancreatic tissue analysis and genotype/phenotype correlation (17). Genetic abnormalities were not detected in the other 15 patients whose MEN-1 clinical syndrome was limited to PHP and a GH-secreting tumor and who did not have a NET. Two additional patients in the Acropara group who were not available to provide DNA samples for research genetic analysis did undergo commercially available screening for MEN-1 and were found to be negative for an *MEN1* mutation. The 2 patients within the Acronet group declined genetic testing (Table 1).

## Discussion

This is the largest series of patients with sporadic PHP and acromegaly yet described. Prior series report PHP in association with pituitary tumors in which genetic testing for MEN-1 was negative (9, 10, 14, 18, 19). However, these reports included a variety of different pituitary tumor types and only a minority of patients with acromegaly. Specifically, in acromegaly cohorts, the most extensive prior study with detailed clinical and genetic evaluations reported 6 patients with both GH-secreting tumors and PHP (8). The prevalence of clinical MEN-1 found among patients with acromegaly reported in this study (6.6%) is consistent with prior reports among all pituitary tumor subtypes, in whom the prevalence of clinical MEN-1 ranges from 4.8% to 7.7% (9, 10). In addition, while most prior reports have evaluated mutations in the *MEN1* gene or *CDKN1B*, this study includes a more comprehensive genetic assessment. While there have been reports of PHP in cohorts with heterogeneous pituitary tumor types (8-10, 20), the prevalence of PHP in a large cohort of patients with acromegaly has not been described previously. Furthermore, this study suggests that the prevalence of positive genetic testing in sporadic PHP and acromegaly is low (6). More specifically, all screened patients having only parathyroid and GH-secreting tumors had negative genetic testing. The single patient within the Acropara group that had a novel genetic finding had a third endocrine tumor (pancreatic NET), which is consistent with a prior study that shows a higher likelihood of positive genetic testing with 3 endocrine tumors rather than 2 (6). In this study, the prevalence of positive genetic testing for *MEN*1 mutations in patients with any 2 sporadic MEN-1-associated tumors was 23 %, but most of the positive genetic testing for *MEN*1 mutations were in patients who had the coupling of parathyroid and pancreatic tumors. In fact, only 2 of the *MEN1* positive genetic tests were in patients with acromegaly (6).

In genetically confirmed autosomal dominant MEN-1, PHP is an earlier and more prevalent clinical finding than other endocrine tumors and is also usually detected at a younger age (1). Conversely, in this study, patients within the Acropara group were older at the time of diagnosis of acromegaly, and in only 9% of them, PHP diagnosis was made before pituitary disease was discovered. This is consistent with a later age of onset described in a cohort of sporadic clinical MEN-1 patients with negative testing for *MEN1* mutations which included all phenotypes of MEN-1, but only few with acromegaly (21). Since younger patients with pituitary tumors, particularly prolactinomas, have a higher prevalence of *MEN1* mutations (7, 22), it might be expected that patients with clinical MEN-1 would also present with pituitary tumors at a younger age. On the contrary, this series demonstrates that, in patients with sporadic PHP and GH-secreting tumors, acromegaly presents later in life.

Since the occurrence of multiple endocrine neoplasia later in life in sporadic cohorts with clinical MEN-1 show that most are genetically negative for mutations in *MEN1* and *CDKN1B* (10, 14, 18, 19), the cause of the coexistence of acromegaly and PHP remains unclear. One possible explanation for this finding is that excess GH could predispose to the development of PHP. In this study, 6.1% of patients with acromegaly were found to have PHP, a prevalence higher than reported in the general population (6.1% vs 0.86%) and also higher than the 1.5% prevalence among patients over 65 years of age (11, 23). However, animal studies show that the administration of GH can alter parathyroid cells, decreasing nucleus/cytoplasmic ratio and impairing their secretory capacity (24). Further evidence against the hypothesis that GH excess leads to PHP includes studies in patients with GH deficiency that have shown that exogenous GH can enhance PTH sensitivity in target organs, increasing calcium levels in plasma and lowering PTH levels (25, 26). Furthermore, reversing GH excess with long-acting somatostatin analogs may be associated with an increase in PTH (27). This evidence decreases the likelihood of a hormonally driven causal relationship between excess GH and PHP and raises the possibility of an underlying unknown genetic predisposition in this group with clinical MEN-1. Alternatively, there may be an ascertainment bias, since patients with asymptomatic PHP may come to attention when seen by an endocrinologist. However, it is also possible that yet-unknown genetic variants predispose to multiple endocrine tumors in these patients. Notably, the presentation of PHP in this group with 56.3% of hyperplastic parathyroid tissue and multiglandular disease in pathology reports is higher than the 10% to 15% reported in sporadic cases (11). The high prevalence of hyperplasia is consistent with the form of PHP among patients with genetically confirmed MEN-1 (1, 11). Regarding adrenal adenomas, although a higher frequency of these tumors was found overall, when analyzing only those patients with available abdominal imaging in the electronic records, the difference becomes a non-significant trend. The more frequent use of abdominal imaging in the Acropara group may explain this finding, as patients with clinical MEN-1 undergo a more thorough search for tumors in other organs and particularly abdominal scans for evaluation of pancreatic NETs associated with this syndrome. Interestingly, only patients within the Acropara group showed the presence of more than one adrenal tumor, which may suggest a genetic predisposition, and further studies are warranted to investigate this.

A higher prevalence of malignant tumors was found among patients within the Acropara group, specifically breast and thyroid cancers (Table 3). The difference between groups remained significant after multivariate logistic regression for overall malignancies and for breast cancer, adjusting for age at last follow-up (*P* = 0.031 and *P* = 0.018, respectively). These findings suggest that the phenotype of genetically negative sporadic acromegaly and PHP is associated with a higher frequency of malignancies, independent of age. Previous publications have shown a higher frequency of breast cancer among female patients with genetically confirmed MEN-1 (28), which may support the idea that there might be a genetic predisposition in patients with acromegaly and PHP with negative genetic testing that involves a similar pathway to the *MEN1* gene. Previous reports in literature have shown a higher prevalence of malignant thyroid neoplasms among patients with acromegaly than in healthy controls, ranging from 0.6% to 10.6% (29, 30), which is consistent with the 2.3% of thyroid carcinoma found among isolated acromegaly patients in this study. Other studies have shown that there is a similar frequency of thyroid malignancies in MEN-1 patients who undergo parathyroidectomy compared to those with sporadic PHP (31). Interestingly, Acropara patients display a significantly higher prevalence of thyroid cancer (13.6%) than that which has been previously published in isolated acromegaly, further suggesting an association between the Acropara phenotype and thyroid malignancies. Furthermore, as a high prevalence of biochemical control was achieved in both groups with and without PHP, and there were no differences in their respective IGF-1 levels at diagnosis or at last follow-up, differences in GH/IGF-1 control did not appear to explain the increase in malignancy rate seen in the group with acromegaly and PHP. In summary, these results suggest an increased risk for malignancies in patients with acromegaly who have PHP, which was not previously known and should therefore be approached accordingly.

One of the main strengths of this study is that the large cohort of well-characterized patients with pathologically confirmed GH-secreting adenomas afforded an ideal group, not only from which to determine the frequency of concurrent PHP, but also to use as a control group of isolated GH-secreting tumors for further distinguishing clinical differences. Unique to this study was not only the detailed clinical phenotype at diagnosis, but also the longitudinal follow-up for clinical outcomes. For example, the finding that there was a similar prevalence of radiation therapy, repeat surgeries and normal IGF-1 at last follow-up suggests that the patients with acromegaly and PHP did not have a more aggressive clinical course than those with isolated GH-secreting tumors. As the coexistence of both conditions is infrequent, the assessment of differences between groups might be underpowered and larger cohorts with this phenotype are needed for establishing definitive associations. In addition, genetic analysis in 16/22 patients within the Acropara group was more comprehensive than in earlier prior reports which were limited to *MEN1* and *CDKN1B* genes. Here, DNA analysis included a full panel of genes, such as *CDC73, CDKN1A, CDKN1B, CDKN2B, CDKN2C*, as well as *AIP*, and evaluation for duplications and deletions using MLPA.

Regarding limitations, it is possible that patients with GH-secreting tumors have parathyroid adenomas or parathyroid hyperplasia incidentally rather than due to a genetic cause, and that what is described here as one clinical entity may have several different causes, including ascertainment bias. In addition, although positive family history of malignancies was found to be more frequent among the Acropara subjects, information on first-degree relatives with cancer was not available for all patients in the isolated acromegaly group. Moreover, patients sent for genetic analysis were interviewed specifically for family and personal history of cancer, favoring a higher rate of ascertainment of malignancy than that available for the retrospective analysis in isolated acromegaly and Acronet patients. Therefore, malignancy history may have been underreported in the isolated acromegaly patients, which could explain the low frequency of breast cancer in this group compared to the general population (32).

While it is known that there is a higher prevalence of colon cancer in acromegaly, patients within the Acropara and isolated acromegaly groups had a high rate of biochemical control (85.3% and 90% normal IGF-1 at last follow-up, respectively) such that the prevalence of colon and other cancers may have been lower than prior reports (33, 34). Also, thyroid cancer might be overrepresented in the Acropara group since thyroid imaging was more frequently performed for the study of PHP, and more incidental thyroid neoplasms might have been diagnosed. However, given the suggestion of a higher rate of malignancy among patients with Acropara with negative genetic testing, it would be prudent to consider earlier and more aggressive cancer screening in these cases.

In conclusion, the phenotype associated with the coexistence of acromegaly and PHP with negative genetic testing might represent a distinct clinical entity not previously well described. Older age at diagnosis of acromegaly, a higher frequency of parathyroid hyperplasia and multiglandular disease rather than adenomas, and the increased frequency of some malignancies represent distinctive features of this novel phenotype. The higher prevalence of malignant neoplasms in this subset of patients with Acropara may indicate a need for more aggressive cancer screening in this population. In addition, the genetic analysis performed in this cohort likely excludes the most frequently attributed genes involved in MEN-1-like phenotypes, including MEN-4. Future studies using whole-genome sequencing may clarify whether yet unidentified genetic mutations are responsible for the clinical manifestations seen in these patients, revealing the mechanisms implicated in this disease.

## Abbreviations

GH: growth hormone
IGF-1: insulin-like growth factor 1
IQR: interquartile range
MEN-1: multiple endocrine neoplasia type 1
MLPA: multiplex ligation-dependent probe amplification
NET: neuroendocrine tumor
PHP: primary hyperparathyroidism
PTH: parathyroid hormone
SD: standard deviation

## Additional Information

***Disclosure Summary:*** L.B.N. has served as a consultant for Ipsen, Pfizer, and has received research funds from Ipsen and Chiasma. L.B.N.’s conflicts were reviewed and are managed by Massachusetts General Hospital and Partners HealthCare in accordance with their conflict of interest policies.

K.K.M. has nothing directly relevant to the manuscript to disclose. She receives study drug at no cost from Pfizer and an investigator-initiated grant and study drug at no cost from Amgen. She has also received study drug at no cost from Marinus and Lawley Pharmaceuticals. She has stock in the following healthcare companies: Amgen, Becton Dickinson, Boston Scientific, Bristol Myer Squibb, and GE. These conflicts were reviewed and are managed by Massachusetts General Hospital and Partners HealthCare in accordance with their conflict of interest policies.

Other authors have no financial interests to declare.

***Data Availability:*** The deidentified datasets generated during and/or analyzed during the current study are not publicly available but are available from the corresponding author on reasonable request.
